# Identification of the effective crane hook's cross-section by incorporating finite element method and programming language

**DOI:** 10.1016/j.heliyon.2024.e29918

**Published:** 2024-04-30

**Authors:** Md Nazmul Hasan Dipu, Mahbub Hasan Apu, Pritidipto Paul Chowdhury

**Affiliations:** a Department of Industrial and Production Engineering, Shahjalal University of Science and Technology, Sylhet, Bangladesh; b Department of Electrical and Electronic Engineering, Sylhet Engineering College, Sylhet, Bangladesh

**Keywords:** Crane hook, Cross-section optimization, Finite element analysis, Curved beam, Numerical stress analysis, Simulation

## Abstract

The crane hook is a widely utilized component in several industries for the purpose of lifting things. The crane hook must possess the capacity to withstand the intended load without encountering any complications, hence ensuring the safety of both personnel and the objects being lifted. The process of analysis is crucial for the effective utilization of a crane hook. The primary aim of this study was to determine the most efficient cross-sectional crane hook among five distinct geometric profiles. This was achieved through the application of finite element analysis using Solidworks software. Subsequently, the identified cross-sectional profile was further examined using the Python programming language, taking into account the classical equation of a curved beam. The five cross-sectional shapes seen in the study were circular, rectangular, trapezoidal, I-shaped, and T-shaped. For the purposes of this investigation, the chosen material for each cross-sectional crane hook model was 34CrMo4 steel. Despite the identical boundary constraints imposed on all the chosen cross-sectional crane hook profiles, it was observed that the trapezoidal cross-sectional crane hook exhibited superior performance compared to the others. The trapezoidal cross-sectional crane hook model exhibited a Von Mises stress of 203 MPa, with a corresponding factor of safety of 3.20. Further experimentation was conducted using Python to examine the trapezoidal profile. The results indicated that an increased level of parallelism in the inner side of the trapezoidal shape corresponded to a higher factor of safety. Hence, it is advisable to maintain the trapezoidal cross-sectional profile of the crane hook, with due consideration given to maximizing the length of the inner parallel side. The enhancement of design leads to a decrease in the likelihood of failure and the occurrence of undesirable accidents.

## Highlights

A crane hook is a component that is frequently used to lift goods. Its optimization and stress analysis are important in order to maintain safety for both life and products. Its optimized cross-sectional profile can provide a better factor of safety under the same boundary conditions like carrying loads. Therefore, its profile optimization is necessary. Highlights of our research follows as.

• Best cross-sectional crane hook's profile identification and a pattern for it.
• Python iterations based on classical equations apart from finite element analysis simulation, to get optimized profile, therefore, this research provide cross-checked evaluation.

## 1 Introduction

### 1.1 Significance of crane hook

In the modern era, cranes are used to move enormous loads from one location to another in numerous industries [1]. The increasing usage of cranes, especially in the construction industry, has been observed in the last couple of years [2]. Besides, a notable portion of the causalities that occurred at construction sites were also discovered to be associated with the operations of cranes [3,4]. A mishandled crane load not only kills individuals but also hampers the critical section of a project [5]. Therefore, it is imperative to perform a comprehensive investigation of the underlying causes of these errors to implement preventive measures to avoid mistreatment [6]. The problems identified in the production lines involving the usage of crane may be attributed to various factors, such as inadequately designed components, improper operation of the crane, or maintenance concerns [4,6,7]. To gain more knowledge about different safety factors pertaining crane components, some researches have already been done [8,9]. And killer hooks [10] — a hook with defective throat latches — were found to be one of the risk factors related to the component of a crane [11]. This finding is supported by the study of King, who found — statistics pertinent to crane accidents from 2004 to 2010 in North America — that the number of accidents due to rigging failure, slings, wire slings, synthetic slings, shackles, hooks, spreader bars, equalizers, no softeners, tag lines, no tag line, tag line tangles, not balance, unknown CG, improper pick points used, unsecured rigging, and wrong rigging type were 5, 3, 1, 2, 0, 2, 0, 0, 1, 2, 2, 0, 8, 1, 4, 5, and 0, respectively. Moreover, apart from these accidental factors, weather conditions were also monitored, and it was stated that of the four weather conditions observed — wind, ice, fog, and lighting — wind was only one causative factor [12]. Although there are some unwanted situations mentioned above relevant to the crane hook, it is highly essential and reliable components commonly employed in industrial environments. However, the compromised reliability of the hook results in unforeseen incidents within the material handling system [13]. Thus, the design of this fixture must comply with the relevant health and safety rules so that the crane can be safely operated and easily maintained by a properly trained operator [[14], [15], [16], [17], [18], [19]]. In addition, safety latches may be incorporated with all hooks to secure the hooks [11]. Furthermore, an assessment of the stress generated by the crane hook is warranted to mitigate the risk of its potential collapse [18,20].

In addition, to alleviate the risk of crane hook-related incidents, several safety regulations suitable to different industrial contexts are recommended by the Occupational Safety and Health Administration (OSHA). Firstly, it is advised that the employer only let personnel who have received training or have expertise operating equipment and machinery [21]. Therefore, industrial hooks should not be utilized without sufficient training. Secondly, slings may not be loaded over their rated capacities [22]. Thus, overloads must be avoided when utilizing a crane hook. Thirdly, whenever any sling is used, several items of it shall be watched, such as damaged, shortened, and kinked, in order to be corrected [23]. Fourthly, never inappropriately attach a hook to a load, and avoid dropped loads and hook damage [24]. Eventually, each day before use, the sling and all fastenings and attachments must be inspected for damage or defects by an experienced individual selected by the employer, and damaged or faulty parts must be removed promptly [25]. So, it is not advisable to use a damaged hook.

### 1.2 Literature review

Several researches have been done to tackle the challenges related to the design of crane hooks. Hadiwidodo et al., analyzed a double crane hook with the help of curved beam theory and finite element analysis. While the stress found theoretically was 73.195 mega Pascal (MPa) the Von Mises stress measured by finite element method was 77.159 MPa. Hence, it was found that the error between the results of the theoretical and numerical calculations was 4.3 % [26].

Sengul and Kam studied developed crane hooks utilized for ship freight and cargo transit. Both experimental and numerical studies were done under 60 kilo Newton (kN) static loading conditions. In the experimental study, ST37 structural steel and Weldox700 high-strength steel were employed to produce the hook. Experimentally, the highest values of stress of the hook fabricated with Weldox700 and ST37 steels under the stress static loading circumstances were obtained as 60 MPa and 57.3 MPa, accordingly. In the numerical analysis, however, the stress value was determined 61.21 MPa. Therefore, compared to the analysis results based on experimental tests and finite element analysis showed that fudge factors in ST37 and Weldox700 steels are 1.8 % and 6 %, respectively. This finding demonstrates that experimental and finite element analysis were in accord with each other and credible [27].

Huang et al., published a research article that described a real-world 4D BIM model built with a tower crane. A novel multi-level elitist genetic algorithm was proposed to determine the optimal lifting path planning and assembly sequence for precast construction [28].

Singh et al., said that determining the optimal material, geometry, and dimensions for certain stress circumstances required a lot more research and investigation. Additionally, it was discovered that a lead and iron alloy with a trapezoidal cross-sectional hook was the most optimal. Furthermore, it was noted that the finite element method has become a vital tool for the design and analysis of these components [29].

A review paper was published by Sadeghi et al., that study identified 59 crane safety risk factors; crane hook was one of them under the term 'crane structural parts' — it was called E10 code on that paper. The findings highlight the importance of crane stakeholders, the environment, and regulatory bodies in a holistic crane safety system. The majority of articles addressed risk factors associated with crane equipment, such as operator aids, blind lifts, safety reliability of crane parts, congested sites, overlapping crane works, cross-operations, and visibility at sites [3].

In their study, Kishore et al., employed a scanning electron microscope to investigate the micro and macro fractography of a 24 T crane hook. Additionally, stress analysis was conducted through the utilization of analytical calculations and finite element modeling utilizing Abaqus® 2018 version. According to the findings, the finite element analysis demonstrated a high level of agreement with the analytical results in the failure zone of the trapezoidal cross section. A proposal was made to develop a rigorous standard for visual examination. The operation of cranes should have been prohibited in instances where they exhibit significant tool marks or notches. If deemed required, it was advisable to conduct appropriate polishing. Additionally, it was proposed that the implementation of ultrasonic testing for crane hooks should be carried out during scheduled shutdowns in order to identify the presence of fatigue cracks [6].

In their study, Pavlovi et al., conducted an analysis and optimization of the geometric properties pertaining to the T-cross section of a crane hook. The computation of maximum stresses at specific locations was performed utilizing the Winkler-Bach theory, where the hook was designed as a curved beam. Both MS Excel and MATLAB® were utilized as software tools. The impact of the geometric constraint on optimization outcomes and cost reductions was evident in the obtained results [30].

The essential sliding angle of the crane hook was investigated by Onur. The study incorporated multiple analytical approaches, including the finite element analysis method, curved beam theory, and simplified theory, to facilitate a comprehensive comparison of simulation outcomes. The drop in safety factor seen at a sling angle of 51° led to the determination that a sling angle of 51° was crucial [13].

The purpose of Desai and Zeytinoglu's study was to optimize the cross section of the crane hook by comparing three different profiles: square, circular, and trapezoidal. The study's findings revealed that a trapezoidal cross section of a hook exhibited superior performance in terms of maximum stress when compared to both circular and square cross sections. The Solidworks® program was employed as a finite element analysis simulation tool [1].

Uddanwadiker conducted a project with the objective of analyzing the stress distribution pattern of the crane hook. This was achieved by the use of the finite element approach, and the obtained results were afterwards validated using the technique of photoelasticity. A finding was made indicating that by extending the region on the inner side of the hook at the point of maximum stress, the level of stress was reduced. From an analytical perspective, it could be observed that the introduction of 3 millimeters increase in thickness resulted in a reduction of strains by around 17 %. Therefore, the design could be modified by augmenting the thickness of the inner curve, so substantially reducing the likelihood of failure. Furthermore, it was proposed that a forging manufacturing technique be employed to produce crane hooks, since that would enable the crane hook to withstand a significant tensile load compared to the casting production process [18].

In their study, Torres et al., conducted an investigation with the aim of determining the factors that contributed to the failure of the crane hook during its operational use. Metallurgical analysis was employed in the investigation of crane hook failures. The failure could be attributed to the development of cracks within the heat-affected zone due to liquification, leading to a brittle fracture. The utilization of the counterfeit crane hook as an indivisible entity was advised [31].

A review paper was published by Neitzel et al., where it was mentioned that crane-related injuries and fatalities were a major concern in the construction industry. It was recommended that injury prevention required adequate training and knowledge of crane safety devices and procedures [4].

In their study, Gough et al., utilized the principles of stress analysis to examine the structural integrity of different cross-sectional designs of crane hooks. A comprehensive explanation is provided on the calculation of bending moment in crane hook loading scenarios, employing the principles of curved beam bending. Furthermore, the utilization of fatigue testing to ascertain the aspect of safety is exemplified. The utilization of photoelasticity has been incorporated alongside the finite element analysis technique in the modeling of crane hooks. The photo elastic method entails fabricating an acrylic copy of the object being investigated and subjecting it to identical loading conditions as the authentic component. Fringe patterns can be observed in specific optical configurations, referred to as polariscopes, due to the optical properties inherent in acrylic materials [32].

### 1.3 Research gap

In the extant literature, distinct geometrical cross-sections of crane hooks were investigated to find the optimal design. However different designs were investigated under different boundary conditions which made it difficult to compare them precisely and find out the optimal design. Again, multiple conceivable variations of dimensions of a particular shape are possible under same boundary conditions. So, an optimal design should consist of not only an optimal geometrical shape but also an optimal dimension for the particular shape. Furthermore, to facilitate in real life scenario, it is necessary to consider the factor of safety — defined as the ratio of ultimate stress to working stress: it shows the component's excess strength over the needed strength to carry that load [[33], [34], [35]] — for each sectional profile. However, a limited number of studies were identified that conducted this comprehensive examination of the specific geometric characteristics of a cross-sectional crane hook.

### 1.4 Objective of this study

The objective of this study is to determine the most efficient cross section for crane hooks among five distinct shapes (circular, rectangular, trapezoidal, I-shaped, and T-shaped) using finite element analysis. Additionally, after obtaining an efficient shape by virtue of the initial finite element analysis, the study aims to conduct a more detailed examination of that shape by varying its several dimensions through iteration with the help of a programming language.

## 2 Methods

### 2.1 Sequence of the study

**Fig. 1:**
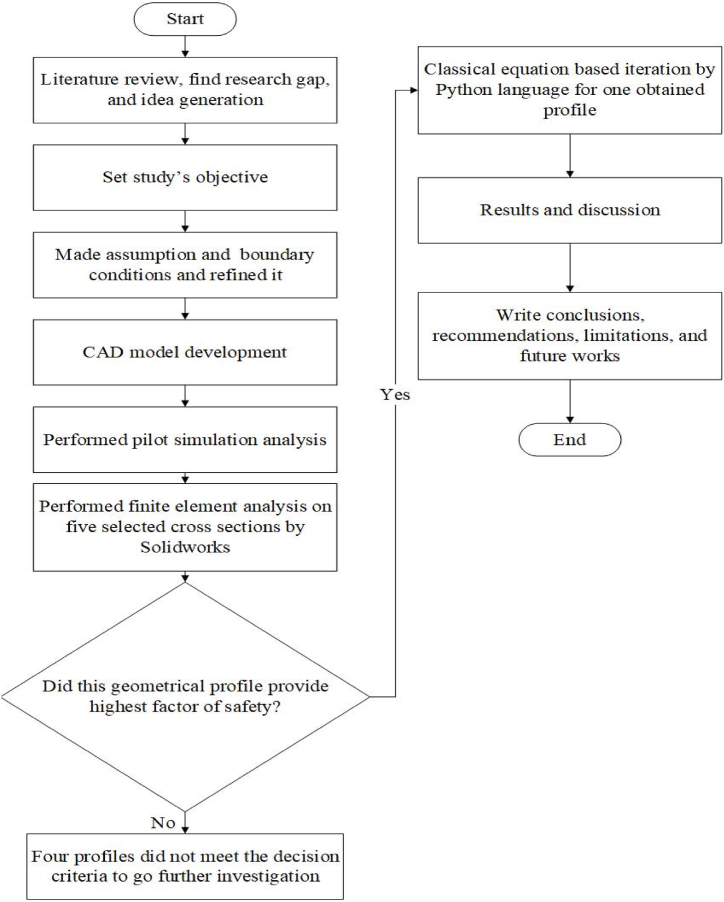
Flowchart of the methodology of this study.

The research commenced by doing a comprehensive evaluation of existing literature, identifying a gap in the current body of knowledge, and subsequently addressing the knowledge gap. Concequently, the research aim was established with the purpose of unearthing the aforementioned objectives. Boundary conditions were taken along with necessary assumptions in light of existing literature. Subsequently, computer aided design (CAD) models were created, followed by the execution of a pilot finite element analysis simulation. This pilot simulation aimed to check the CAD models as well as assess the selected boundary conditions. Eventually, finite element calculations were conducted on five distinct cross-sectional profiles of crane hooks — circular, rectangular, trapezoidal, I-shaped, and T-shaped profiles. The particular shape which had the highest factor of safety compared to other shapes, that shape was subjected to additional analysis using the Python programming language, based on classical equations. This analysis involved testing alternative sizes of that shape, while maintaining a constant profile area. Thenceforth, the findings and subsequent discourse were formulated in accordance with the analysis conducted. However, it is important to acknowledge the limits of this study, which may have impacted the results. In light of these limitations, future research should be conducted to address these gaps and build upon the current findings. Fig. 1 illustrates all of these sequences of the study.

### 2.2 Boundary conditions and assumptions

Finite element models need the formulation of mathematical assumptions for the depiction of the geometry and behavior of the designed components [36]. To accurately compare all five cross-sectional crane hooks in this study, the following assumptions and boundary conditions needed to remain the same for all of those crane hook models.

a) Das et al., did a failure analysis of the crane hook by letting 4 tons (40 kN almost) of constant force [37] as well as Onur considered 40 kN force for crane hook analysis [38]. Similarly, Fetvaci et al., analyzed a simple crane hook by finite element analysis under consideration of 5 tons (50 kN almost) force [39] and Krishnaveni et al., also performed a static analysis of crane hook T section by applying 6 tons (60 kN almost) force [40]. In this study, an external constant downward force of F = 50 kN was considered.
b) In addition, the force acts in real the world between the contact surface of the rope, which goes throughout the crane hook, and the crane hooks inside surface. This contract surface is a minute area. So, force should have to be applied on a small load carrying surfaces during the finite element analysis [33,38,41]. Therefore, the inside surface of each design crane hook was split into a little portion at the portion where the load was applied.
c) Cross-section areas should be designed in such a way that each one has the same amount of area. A rounded 2827 square millimeters area was chosen for all of those cross sections. So, the same amount of cross-sectional area helped to make a logical comparison of all this study's crane hooks.
d) Having an inside radius of 50 millimeters for all selected crane hooks.
e) Having an outside radius of 110 millimeters for all selected crane hooks.
f) 34CrMo4 is a strength class T of the crane hook [13]. Hence, all the models in the study had the same material, which was 34CrMo4. Because it has better mechanical properties which is suitable for crane hook.
g) The same fine mesh was applied for each model.

### 2.3 Five selected cross sections

**Fig. 2:**
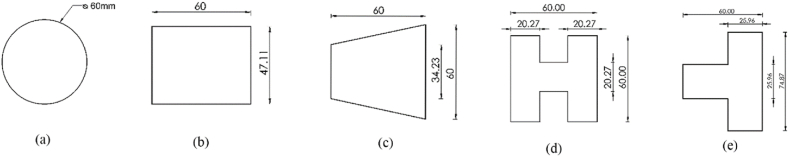
Dimension of cross-sectional profiles of the crane hooks.

For the simulation investigation, five CAD models of various crane hooks were created: circular, rectangular, trapezoidal, I-shaped, and T-shaped. After rounding off, each of those crane hook models had a cross-sectional area of 2827 square millimeters. Fig. 2a–e depicted the selected cross-sectional areas. Here each of these profiles has 60 millimeters gaps between two faces so that the constant boundary condition can be maintained—the difference between curved beams inside radius 50 millimeters and outside radius 110 millimeters is 60 millimeters.

### 2.4 Material selection for this study

If a crack develops in the crane hook, it can cause a fracture of the hook and lead to a serious accident. So, the right material section for a crane hook is important [13]. If too brittle material is chosen, it will fail suddenly during the work after a crack develops. Because in brittle fractures, there is sudden propagation of the crack, and the hook fails suddenly. This type of fracture is very dangerous because it is difficult to detect. So, workers at that time would not have had a moment to move on. So, sudden failure should be avoided by not choosing too brittle material. On the other hand, the crack propagates continuously in a ductile fracture, is more easily detectable, and is hence preferred over a brittle fracture [18]. But too much ductile material will not be able to sustain a crane hook's activities. So, a material might be chosen that is neither too brittle nor too ductile. Therefore, it is vital to scrutinize its mechanical behavior like Young's modulus, mass density, Yield strength, Poisson's ratio, and so on [42]. In order to select perfect material for crane hook, there are several material classes and crane hook's minimum yield strength is related to five strength classes (M, P, S, T, V). For instance, strength class-T denotes structural low alloy steel such as 34CrMo4 [13,43] which was chosen for this study for its high lasting strength and creep strength at high temperatures, good impact toughness at low temperatures, good hardenability, and medium machinability.

**Table 1:** Material properties of 34CrMo4 steel.

Properties	Value
Elastic modulus	210000 MPa
Poisson ratio	0.28
Mass density	7800 kg/m^3^
Tensile strength	900 MPa
Yield strength	650 MPa

Material properties of 34CrMo4 steel, shown in Table 1, have been taken from the Solidworks® materials library. All of these properties of 34CrMo4 were incorporated into the particular crane hook CAD model by applying 34CrMo4 material from the Solidworks® standard material library during the finite element analysis. Here, the yield strength of 34CrMo4 is 650 MPa which is vital for determining factor of safety.

**Table 2:** Mesh properties.

Matrices	Value
Mesh type	Solid mesh
Mesh density	Fine
Mesh parameters	Standard mesh
Jacobian points	4 points

A mesh is a subdivision of a domain into finite elements that are used to approximate the outcome [44]. In other words, it is an automatic volumetric discretization of the 3D model in order to achieve an approximate solution [45]. In this simulation study, the mesh information, shown in Table 2, was taken from Solidworks®. The status of mesh density for this study's models was fine. By default, Solidworks® mesh density status is in the middle between the leftmost position (Coarse) and the rightmost position (Fine). By putting the density status slider from the middle to the fine, it decreases the mesh size by half the default, thus increasing the analysis time and accuracy of the outcome. In this study, all of the models witnessed the corresponding possible smallest mesh size (Fine) in light of their geometry. Additionally, mesh parameters were kept as standard mesh.

### 2.5 Finite element method

The finite element method is a numerical method of developing a model of a structure and analyzing its mechanical properties. It allows the application of a desired force at a desired location to analyze related stress and strain in any shape of material [46,47]. In other words, the finite element method is a computational technique employed to investigate sophisticated structures and continua that is beyond the capabilities of conventional analytical approaches [48]. The approach is widely used in engineering and mathematical modeling [49]. The results of finite element analysis are sufficiently precise for engineering applications and can be obtained at acceptable expenses. Finite element analysis is used in stress analysis, heat conduction, or various other domains. Finite element method first began to be used in orthopedic biomechanics in the 1970s to assess the stresses experienced by human bones under functional loading conditions. In the 1980s, there was a notable increase in the use of finite element analysis in dentistry. This focus was mostly on design and the analysis of stresses and deformations caused by functional load factors [48]. The finite element approach has been frequently employed in the analysis of components because of the tremendous strength of the technology [36], because the solutions of finite element analysis were reliable and near enough to the outcomes acquired from experimental tests [27]. As a result, due to its accuracy and repeatability, finite element analysis is used in mechanical and industrial perspectives, anthropology, electronics, biomedical, and material science [50,51]. In recent years, a surge in the use of finite element analysis has been observed. For instance, used finite element analysis to find out the optimal design of an artificial hip implant with the least linear wear rate at the trunnion junction [52]. Similarly, utilized finite element analysis to find out the best material for hip stems with the least wear rate at the inter-contact regions of the acetabular cup [53]. Again, tried to get the best combination of materials, shapes, and profiles for hip implants incorporating the finite element analysis [54]. Moreover, the reliability of the ultra high molecular weight polyethylene (UHMWPE) as the constituent material for an acetabular cup was tested by using this technique [55]. On the other hand, proposed a safer and more economical design of machine shafts by analyzing their fatigue life using finite element analysis [56].

### 2.6 Tools and technique

There were some previous works where Solidworks® was used in order to perform finite element analysis [1,26]. Therefore, Solidworks® 2020 was employed for both the CAD model development and the finite element analysis. Furthermore, the Python programming language was used to do iterations on the basis of curve-beam equations.

Four stress analysis techniques are Max Von Mises stress, Max Shear stress (Tresca), Mohr-Coulomb stress, and Max Normal stress. Among those Max Von Mises stress technique is considered the best predictor of actual failure of ductile materials (aluminum, steel, bronze, brass, etc) and provides a good indication of true factor of safety. Another technique namely Max Shear stress (Tresca) is also applicable for ductile materials but its result is less accurate. However, both Mohr-Coulomb stress and max normal stress are suitable for brittle materials (glass, cast iron, etc). Max Normal stress is the least accurate technique among all [33]. Therefore, Max Von Mises stress technique was chosen for this study in light of 34CrMo4 material properties. In favor of this Von Mises stress technique, several studies relied on this technique to analyze the stress on crane hooks [1,26]. Additionally, this technique is applied in other fields where Von Mises is appropriate for the finite element method. For instance, Goktas et al., used Von Mises as a finite element analysis technique to find out the best design of the hip implant among three different shape designs — elliptic, oval, and trapezoidal— as well as the same dimensions and material combinations under static loading [42]. Another example is Celik et al.'s work, which attempted to analyze the performance of polylactic acid as a liner in total hip arthroplasty using the finite element method. Some factors, such as von Mises stress, total deformation, and factor of safety, were implemented using five distinct models. In light of the analysis, it was concluded that the best mechanical performance of polylactic acid was found with Al-Zi [57].

### 2.7 Curve beam equations for python code

Curved beams are known to transfer loads more efficiently than straight beams [58]. Crane hooks are one of the mechanical elements that appear in the shape of curve beams, written by Nudehi and Steffen, in chapter two of the book entitled Analysis of Machine Elements Using Solidworks® Simulation 2021 [33]. The curved beam's classical equations [1,18,33,59] are mentioned below which were used to write Python programming code.

(i) σ=±bendingstress±axialstress

In equation (i), ***σ*** represents the curved beam's stresses which is a resultant of bending stress and axial stress. ‘Plus’ symbol indicates the tensile stress, on the other hand, ‘Minus’ symbol represents the compressive stress. Stress at inside of a curved beam can be calculated based on following equation (ii).

(ii) $$\sigma_{i}=\frac{MC_{i}}{AeR_{i}}+\frac{F}{A}$$

here, ***σ***_***i***_ is the inside stress. The signs of ***M, C***_***i***_, ***A***, ***e***, ***R***_***i***_, and ***F*** are bending moment, distance from the neutral axis to the inside surface, cross-sectional area of the beam, distance between the centroidal axis and neutral axis, radius to inside (concave surface), and applied vertical force, respectively. However, stress at outside should be determined by equation (iii) which is mentioned below.

(iii) $$\sigma_{o}=-\frac{MC_{o}}{AeR_{i}}+\frac{F}{A}$$

In the equation (iii), ***σ***_***o***_ represents the outside stress of the curved beam. Here, ***C***_***o***_ and ***R***_***o***_ represent distance from the neutral axis to the outside surface, and radius to outside (convex surface), respectively.

(iv) $$R_{o}=R_{i}+h$$

In the equation (iv), h is the width of beam cross-section which was height of the trapezoidal in this study. Additionally, the value of this h can be obtained from following equation (v).

(v) $$h=R_{o}\;–\;R_{i}$$

The area of the trapezoidal cross section can be determined by following equation (vi), where ***S***_***i***_ and ***S***_***o***_ are the inside and outside parallel sides’ length of this trapezoidal profile.

(vi) $$A=\frac{1}{2}\times \left(S_{i}+S_{o}\right)\times h$$

Radius to centroid of beam (***R***_***c***_) and radius to the neutral axis (***R***_***n***_) can be calculated by following equations (vii) and (viii), respectively.

(vii) $$R_{c}=R_{i}+\frac{hS_{i}+2S_{o}}{3S_{i}+S_{o}}$$

(viii) $$R_{n}=\frac{A}{S_{o}-\;S_{i}+\frac{\left(S_{i}R_{o}-\;S_{o}R_{i\;}\right)}{h}\times \ln\;\left(\frac{R_{o\;}}{R_{i}}\right)}$$

Distance from the neutral axis to the inside surface (***C***_***i***_), distance from the neutral axis to the outside surface (***C***_***o***_), and distance between the centroidal axis and neutral axis (***e***) can be found by following equations (ix), (x), and (xi), respectively.

(ix) $$C_{i}=R_{n}\;-\;R_{i}$$

(x) $$C_{o}=R_{o}-R_{n}$$

(xi) $$e=R_{c}-R_{n}$$

Bending moment ***M*** can be calculated by following equation (xii) where ***D***_***h***_ indicates horizontal distance between the vertical line along which force is applied and the centroidal axis.

(xii) $$M=F\times D_{h}$$

The factor of safety's equation is mentioned below [33]. In this equation (xiii), strength is the yield strength of the 34CrMo4, which is 650 MPa (it was also used to write Python language code), and stress is the maximum Von Mises stress that the crane hook CAD model witnessed due to the boundary conditions.

(xiii) $$Factor\;of\;safety=\frac{strength}{stress}$$

**Fig. 3:**
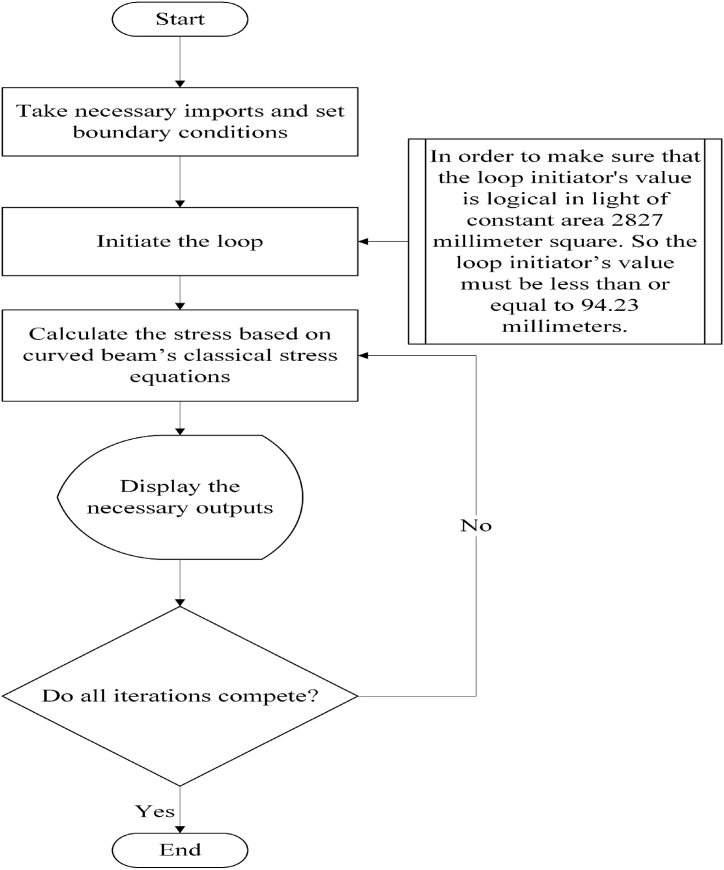
Python code executing flowchart.

The flowchart depicted in Fig. 3 shows how the aforementioned equations were implemented in Python codes. Only the trapezoidal shape required the use of this specific set of code and calculations. When compared to other geometric profiles, the trapezoidal cross section offered the highest safety factor. As a result, it became the subject of further research.

In the detailed research of the trapezoidal cross-sectional profile, consistent boundary constraints — 2827 square millimeters area of the trapezoidal profile, 60 millimeters of height of trapezoidal parallel sides, 50 millimeters of inside radius of the curved beam, and 50 kN of external vertical force — were assigned to corresponding variables in the Python code (Appendix-A). After that, an initial value was needed for the Python loop. As the goal of this Python based iteration was to determine how the factor of safety — which was the criterion for the identification of the best profile dimension — was influenced by the diverging profile dimension of a trapezoidal profile, even though all of the profiles were trapezoidal and underwent constant boundary conditions, so the initial value of the Python loop must come from the trapezoidal profile dimension. Because this profile dimension was subject to change. As the inside and outside radii of the curved beam were 50 millimeters and 110 millimeters in sequence, aforementioned in boundary conditions, thus these two parameters were kept to be constraint. On account of that, only two parameters of the trapezoidal geometry could be altered: inside and outside parallel side lengths. Between these two parameters, the outside parallel side length was considered for the loop initial value, and another one was left for having a corresponding value in order to maintain 2827 square millimeters area. Now the question was: what would be that value, and what would be the loop limitation? As it was known, the area of each cross-section should be 2827 square millimeters, and the distance between two parallel sides was 60 millimeters (because this distance was the difference between the inside and outside radii); therefore, the sum of the two parallel sides of the trapezoidal profile had to be 94.23 millimeters (rounded off to hundredths) — obtained by following a simple calculation.

$$\Rightarrow \frac{1}{2}\times \left(inside\;parallel\;side+outside\;parallel\;side\right)\times height=area\;of\;trapezoidal\;profile$$

$$\Rightarrow \left(inside\;parallel\;side+outside\;parallel\;side\right)=\frac{2\times area}{height}$$

$$\Rightarrow \left(inside\;parallel\;side+outside\;parallel\;side\right)=\frac{2\times 2827}{60}$$

⇒(insideparallelside+outsideparallelside)=94.23millimeters

So, the loop initiator might have had the maximum value of 94.23 millimeters for its outside parallel side, but it was not logical because in this case, the inside parallel side would be 0 millimeters. Hence, 90 millimeters of the outside parallel side was given as the Python loop's initial value, and the loop was continued for every 10 millimeters successive decrease of the outside parallel side. As a result, several variations of this cross-section were used to get the respective stress and factor of safety while preserving the same boundary constraints. To put it another way, both the inside and outside parallel sides of the trapezoidal profile were altered, resulting in nine alternative iterations for this profile with the help of the Python language. The findings of those iterations are listed under the analysis portion of this study, and they are illustrated visually in the conclusion section.

**Fig. 4:**
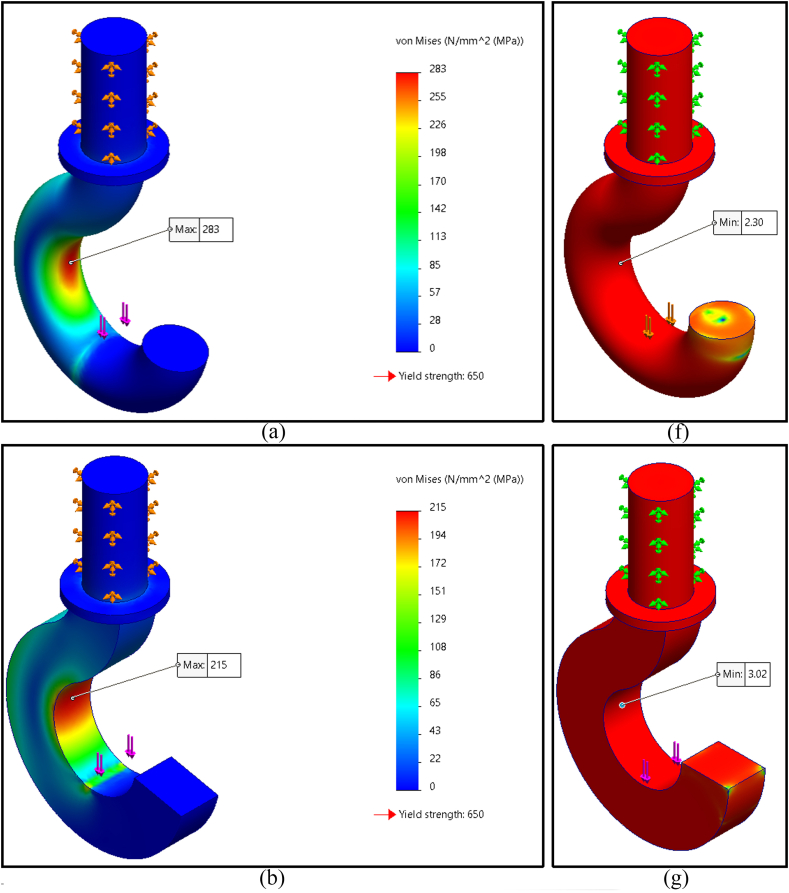

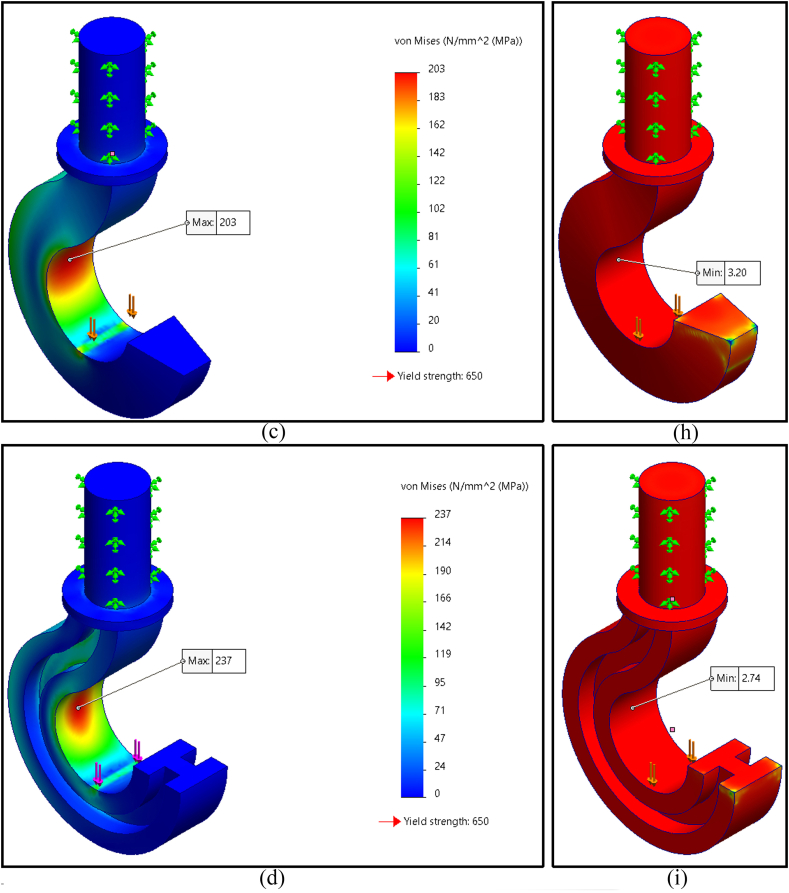

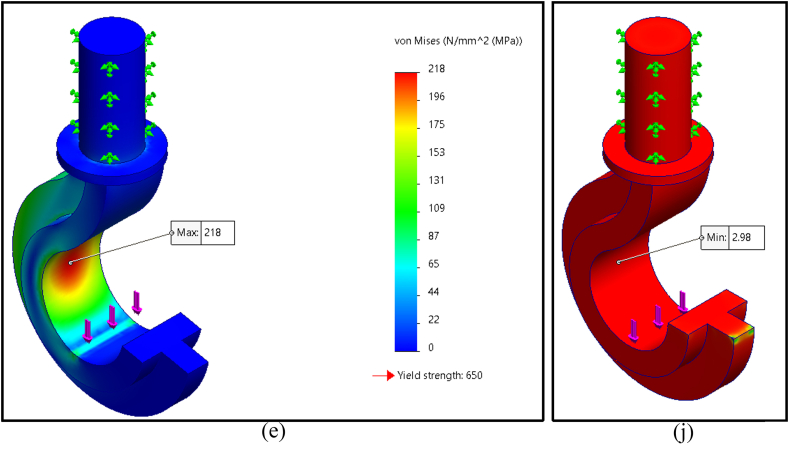
Simulation results of Von Mises stress and factor of safety for five different cross sectional crane hooks.

## 3 Analysis and findings

### 3.1 Finite element analysis

The finite element analysis results of maximum Von Mises stress for five distinct geometrical cross sections of the crane hook are shown in Fig. 4a–e; these cross sections were circular, rectangular, trapezoidal, I-shaped, and T-shaped profiles. The maximum magnitude of Von Mises stresses on circular, rectangular, trapezoidal, I-shaped, and T-shaped profiles was found to be 283 MPa, 215 MPa, 203 MPa, 237 MPa, and 218 MPa (rounded off), respectively. Among those, the trapezoidal profile had the lowest maximal Von Mises stress.

The simulation results also indicated the factor of safety of the five cross-sectional crane hook profiles shown in Fig. 4f–j. The factor of safety of circular, rectangular, trapezoidal, I-shaped, and T-shaped profiles was determined to be 2.30, 3.02, 3.20, 2.74, and 2.98 (rounded off to hundredths). The trapezoidal profile had the highest safety factor of all. Therefore, the best crane hook's cross-sectional geometry is trapezoidal shape. Table 3 shows the summary of the finite element analysis results.

**Table 3:** Simulation results summary of all five selected cross-sections.

Cross section	Von Mises stress	Factor of safety
Circular	283 MPa	2.30
Rectangular	215 MPa	3.02
Trapezoidal	203 MPa	3.20
I-shaped	237 MPa	2.74
T-shaped	218 MPa	2.98

### 3.2 Programming language based iterations

Albeit it was found from the finite element analysis that the trapezoidal cross section was the best one of all, several trapezoidal shapes were possible under same boundary conditions of 2827 square millimeters and a height of two parallel sides of 60 millimeters. So, more investigation was needed. The iteration for the several trapezoidal profiles was done in light of the Python code mentioned in Appendix-A while maintaining the same boundary conditions. These results were put in Table 4.

**Table 4:** Python iterations based on classical curve beam equations.

Iterations	Inside parallel side (millimeters)	Outside parallel side (millimeters)	Parallel distance (millimeters)	Area (square millimeters)	Maximum stress (rounded off MPa)	Factor of safety
1	4.23	90	60	2827	429	1.52
2	14.23	80	60	2827	333	1.95
3	24.23	70	60	2827	276	2.35
4	34.23	60	60	2827	239	2.72
5	44.23	50	60	2827	213	3.05
6	54.23	40	60	2827	195	3.33
7	64.23	30	60	2827	183	3.55
8	74.23	20	60	2827	176	3.69
9	84.23	10	60	2827	174	3.73

It needs to be mentioned that the results of finite element analysis and classical equation-based analysis are different even when the same input values are given. In addition, Onur also showed that finite element method and curved beam theory-based result of maximum stress were different [38]. But, as Nudehi, S. S., and Steffen, J. R. wrote in chapter two of the book Analysis of Machine Elements Using Solidworks Simulation 2021, the finite element analysis method gives a more accurate solution than that of the classical method does [33]. Thus, albeit iteration number 4 had the same boundary conditions in both methods, — finite element analysis and the classical method — the results were different.

The deliberation of using the Python tool and classical equations on the earlier obtained trapezoidal profile by finite element analysis was to do many iterations to find a pattern on how the trapezoidal profile was germane to the factor of safety: so, finding a pattern was the top priority. Table 4 shows that the factor of safety — which was the criterion for the identification of the best profile dimension — gradually increased when the outside parallel side of the trapezoidal profile was subjected to a decrease: both variables were inversely proportional to each other. Therefore, the less the outside parallel side of the trapezoidal profile, the greater the factor of safety it gives, while boundary conditions were the same. It is shown visually in the conclusion section.

**Fig. 5:**
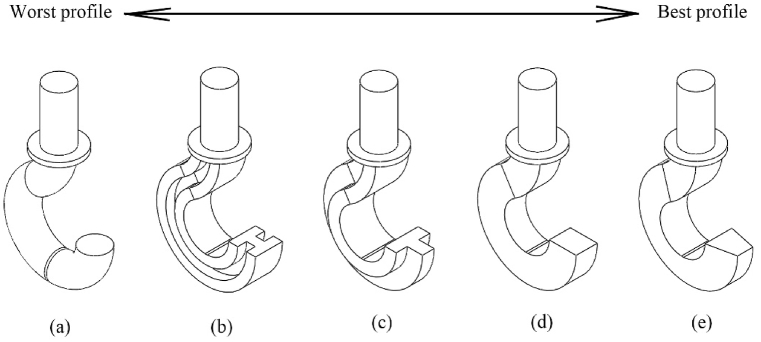
Comparison of different standard geometrical profiles based on factor of safety.

## 4 Conclusions

### 4.1 Summery of the study

Based on the analysis and findings, it is found that a crane hook with a trapezoidal cross-sectional shape offers the utmost degree of safety depicted in Fig. 5a–e. Although all of these cross-sectional crane hooks share the same boundary conditions, it is evident that the trapezoidal cross-sectional crane hook exhibits superior performance compared to the other designs in light of factor of safety. In addition, the trapezoidal cross-sectional hook experiences the lowest level of stress when subjected to an external load. Thus, it is concluded that the utilization of a trapezoidal cross section in the construction of the crane hook is a favorable option due to its ability to enhance the factor of safety and mitigate the likelihood of accidents.

**Fig. 6:**
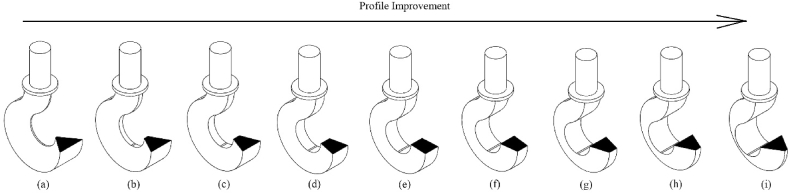
Profile shape improvement from left to right.

It is more appreciated to keep the inside parallel side length of the trapezoidal profile as long as possible. Because it improves the profile of the design in light of safety. Fig. 6a–i express this idea where Fig. 5a represents the profile of iteration-1 in Table 4. Similarly, Fig. 6b–i indicate the profiles of iterations of 2–9 respectively.

### 4.2 Recommendations for practical implementation

a) Design a crane hook with a trapezoidal cross-sectional profile in order to improve safety.
b) Keep the outside parallel side as small as possible to make better cross section. It can be inferred from Fig. 6i that a trapezoidal profile whose outside parallel side is near zero becomes a triangular profile, which is the best one.

### 4.3 Limitations and future works

One limitation of the study was that it did not take into account the presence of a fillet radius. However, it is important to note that, in practice, the removal of sharp edges is necessary to ensure the comfort of the rope or chain that passes through the crane hook. Moreover, no experimental tests could be conducted which was another limitation of this research.

In future research, it might be prudent to investigate incorporating a rational fillet radius. In addition, sensitivity analysis may be performed to figure out which independent variables — variation of loads and cross-sectional area — have the abound influence on the dependent variable of factor of safety. Furthermore, this sensitivity analysis might be conducted with a double crane hook instead of a single crane hook. Additionally, since the crane hook is manufactured by either a drop forged or hammer forged manner [13], the challenges during the manufacturing and operational phases can also be analyzed in the future study.

## Data availability

The CAD models can be given upon request.

## Funding

The authors did not receive any founding or support from any organization or institution.

## CRediT authorship contribution statement

**Md Nazmul Hasan Dipu:** Writing – original draft, Software, Methodology, Formal analysis, Conceptualization, Writing – review & editing. **Mahbub Hasan Apu:** Visualization, Software. **Pritidipto Paul Chowdhury:** Writing – review & editing, Supervision, Writing – original draft.

## Declaration of competing interest

The authors declare that they have no known competing financial interests or personal relationships that could have appeared to influence the work reported in this paper.

## Appendix A Supplementary data

Python Code Python Code
