# Supplementary material for: Identification of the effective crane hook's cross-section by incorporating finite element method and programming language
Source: Heliyon. 2024 Apr 30;10(9):e29918. doi: 10.1016/j.heliyon.2024.e29918 (PMC11101710; doi:10.1016/j.heliyon.2024.e29918)
Supplement: Python Code [file mmc1.docx]

**APPENDIX-A**

| **1**  **2**  **3**  **4**  **5**  **6**  **7**  **8**  **9**  **10**  **11**  **12**  **13**  **14**  **15**  **16**  **17**  **18**  **19**  **20**  **21**  **22**  **23**  **24**  **25**  **26**  **27**  **28**  **29**  **30**  **31**  **32**  **33**  **34**  **35**  **36**  **37**  **38**  **39**  **40**  **41**  **42**  **43**  **44**  **45**  **46**  **47**  **48**  **49**  **50**  **51**  **52**  **53**  **54**  **55**  **56**  **57**  **58**  **59**  **60**  **61**  **62**  **63**  **64** | **#--------------------------------------------------------------------------------Description**  **#This program is about determination of the stress of trapizodal crane hook under the present study's boundary conditions.**  **#Units are millimeter (mm) and netwon (N).**  **#As both profile area 2827 mm square and parallet sides distant 60mm are constant, the sum of both parallel sides must be (2827*2)/60 = 94.23mm almost. So, neither any parallel side can be larger than 94.23mm.**  **#--------------------------------------------------------------------------------Abbreviations**  **#A = Area of trapezoidal profile**  **#h = Height of trapezoidal parallel sides**  **#F = Applied vertical force**  **#Ri = Inside radius of curved beam**  **#Ro = Outside radius of curved beam**  **#Si = Inside parallel side length**  **#So = Outside parallel side length**  **#Rc = Centroid_radius**  **#Rn = Neutral_radius**  **#Ci = Distrance between neutral axis to the inside surface**  **#Co = Distrance between neutral axis to the outside surface**  **#e = Distrance between centroid axis to neutral axis**  **#Dh = Horizontal distance between the vertical line along which force is applied and the centroidal axis**  **#M = Moment**  **#inside_stress = Classical equation for calculating stress at the inside concave surface.**  **#outside_stress = Classical equation for calculating stress at the outside convex surface.**  **#FoS = Factor of safety**  **#--------------------------------------------------------------------------------Standard Imports**  **import math**  **#--------------------------------------------------------------------------------Set Boundary Conditions**  **A = 2827 #millimeters**  **h = 60 #millimeters**  **Ri = 50 #millimeters**  **F = 50000 #Netwon**  **#--------------------------------------------------------------------------------Calculations**  **So = 90 #millimeters #initiate the value**  **print("{:<20} {:<25} {:<25} {:<20} {:<8} {:<17} {:<17}".format("Iteration number", "Inside parallel side", "Outside parallel side", "Parallel distance", "Area", "Maximum stress", "Factor of safety"))**  **for i in range(1,10):**  **Si = (float(A)*2/float(h)) - float(So) #millimeters**  **Ro = float(Ri)+float(h) #millimeters**  **Rc = float(Ri)+(float(h)*(float(Si)+2*float(So)))/(3*(float(Si)+float(So))) #millimeters**  **Rn = float(A)/(((float(Si)*float(Ro)- float(So)*float(Ri))/float(h))*math.log(float(Ro)/float(Ri),2.7182818284590452353602874713527) - (float(Si)-float(So))) #millimeters**  **Ci = float(Rn) - float(Ri) #millimeters**  **Co = float(Ro) - float(Rn) #millimeters**  **e = float(Rc) - float(Rn) #millimeters**  **Dh = float(Rc) #millimeters**  **M = float(F)*float(Dh) #newton-millimeters**  **inside_stress = (float(M)*float(Ci))/(float(A)*float(e)*float(Ri)) + (float(F)/float(A)) #newton-millimeters square**  **outside_stress = -(float(M)*float(Co))/(float(A)*float(e)*float(Ro)) + (float(F)/float(A)) #newton-millimeters square**  **if abs(float(inside_stress)) >= abs(float(outside_stress)):**  **FoS = 650000000/(1000000*float(inside_stress)) #multiple by 1000000 in order to make the same unit "pascal"** **but FoS itself is unitless**  **print("{:<20} {:<25} {:<25} {:<20} {:<8} {:<17} {:<17}".format(i, round(Si,2), round(So), h, A, round(inside_stress), round(FoS,2))) #newton-millimeters square**  **So = float(So) - 10 #millimeters**  **else:**  **FoS = 650000000/(1000000*float(outside_stress)) #multiple by 1000000 in order to make the same unit "pascal"**  **print("{:<20} {:<25} {:<25} {:<20} {:<8} {:<17} {:<17}".format(i, round(Si,2), round(So), h, A, round(outside_stress), round(FoS,2))) #newton-millimeters square**  **So = float(So) - 10 #millimeters** |
| --- | --- |
